# Prenatal Diagnosis of Mosaic Tetrasomy 18p in a Case without Sonographic Abnormalities

**Published:** 2017-01-17

**Authors:** Javad Karimzad Hagh, Thomas Liehr, Hamid Ghaedi, Mir Majid Mossalaeie, Shohreh Alimohammadi, Faegheh Inanloo Hajiloo, Zahra Moeini, Sadaf Sarabi, Davood Zare-Abdollahi

**Affiliations:** 1 *Parseh Pathobiology & Genetics Laboratory, Tehran, Iran.*; 2 *Jena University Hospital, Friedrich Schiller University, Institute of Human Genetics, Jena, Germany, * *Iran.*; 3 *Department of medical Genetics, Faculty of Medicine, Shahid Beheshti University of Medical Sciences, Tehran, Iran.*; 4 *Endometrium and Endometriosis Research Center, Faculty of Medicine, Hamedan University of Medical Sciences, Hamedan, Iran.*; 5 *Genetics Research Center, University of Social Welfare and Rehabilitation Sciences, Tehran, Iran.*

**Keywords:** Tetrasomy, prenatal, cordocentesis, amniocentesis, polydactyly, isochromosome 18p, marker, small supernumerary marker chromosome (sSMC)

## Abstract

Small supernumerary marker chromosomes (sSMC) are still a major problem in clinical cytogenetics as they cannot be identified or characterized unambiguously by conventional cytogenetics alone. On the other hand, and perhaps more importantly in prenatal settings, there is a challenging situation for counseling how to predict the risk for an abnormal phenotype, especially in cases with a* de novo* sSMC. Here we report on the prenatal diagnosis of a mosaic tetrasomy 18p due to presence of an sSMC in a fetus without abnormal sonographic signs. For a 26-year-old, gravida 2 (para 1) amniocentesis was done due to consanguineous marriage and concern for Down syndrome, based on borderline risk assessment. Parental karyotypes were normal, indicating a *de novo* chromosome aberration of the fetus. FISH analysis as well as molecular karyotyping identified the sSMC as an i(18)(pter->q10:q10->pter), compatible with tetrasomy for the mentioned region. Cordocentesis was done due to normal sonography and the results from amniocentesis were confirmed. The parents opted for pregnancy termination and post mortem examination now noted, low anterior hairline, large philtrum, low-set posteriorly rotated malformed ears with prominent antihelix, lower limbs joint contracture and digital anomalies, including long and narrow toes with clinodactyly of the 1^st^ and 5^th^ toes and postaxial polydactyly of one hand. *De novo* i(18p) can be considered as a special case in the sense that the major relevant phenotypes mentioned for it, i.e. feeding difficulties, abnormalities in muscle tone and developmental/mental retardation, cognitive and behavioral characteristics, recurrent otitis media and seizures, are mostly postnatal. This emphasizes the necessity to determine the nature of a *de novo* euchromatic marker chromosome, especially in cases with normal ultrasound result and the suitability of a cordocentesis in order to better predicting the pregnancy outcome and parental counseling.

Small supernumerary marker chromosomes (sSMC) are still a major problem in clinical cytogenetics as they cannot be identified or characterized unambiguously by conventional banding cytogenetics alone. Consequently, there is a challenging situation for counseling how to predict best the risk for an abnormal phenotype, especially in *de novo* cases. Approximately one third of the sSMC cases are associated with specific clinical syndromes, like the i(18p)- or tetrasomy 18p-syndrome (OMIM: 614290), the der(22)t (11;22)- or Emanuel- syndrome (OMIM: 609029), the i(12p)- or Pallister Killian- syndrome (OMIM: 601803), the inv dup(22)- or Cat-eye-syndrome (OMIM: 115470), or the inv dup(15)- syndrome (1-3). Even though recently some more syndromes could be characterized (3-4), the majority of sSMC have not yet been correlated with distinctive clinical syndromes (2, 3). According to Liehr (3) the four chromosomes most often involved in sSMC formation are #15 (~26%), #22 (~17%), #12 (~11.5%) and #18 (~7.5%).

Isochromosome 18p has an approximate prevalence of 1/140,000 to 180,000 live births and thus is a relatively rare chromosomal aberration; still it appears to be one of the most commonly observed isochromosomes (5, 6). It has been proposed that isochromosome 18p arises as a result of two independent events: nondisjunction and centromeric misdivision based on the fact that all cases reported thus far have been monocentric (7-9). As major mechanisms, it is suggested that nondisjunction occurs during maternal meiosis II followed by centromeric misdivision, implying that maternal age may play a role in the formation of the isochromosome (1, 9).

Based on the largest series of individuals with tetrasomy 18p described to date, tetrasomy 18p syndrome is characterized by nonspecific morphologic features; developmental delay and subsequent low birth weight, microcephaly, hypotonia, low- set ears, strabismus, scoliosis/ kyphosis, foot anomalies and cryptorchidism. Feeding difficulties, abnormalities in muscle tone, developmental/ mental retardation, recurrent otitis media and seizures are also observed while cardiac and renal malformations are rare (5). However, as for most genetic disorders, the phenotypic spectrum of tetrasomy 18p is wide. This holds true the more for mosaic cases, which can vary from an apparently normal phenotype, to multiple abnormalities mentioned earlier. Here, we present our experience of prenatal diagnosis of a mosaic tetrasomy 18p case showing no obvious sonographic signs or symptoms.

## Case report

A 26-year-old, gravida 2 (para 1) was referred for amniocentesis at 16 weeks of gestation because of her own personal request; the latter was justified by a concern for Down syndrome based on borderline maternal serum screening risk assessment and consanguineous marriage. Anomaly scan ultrasound and echocardiography were unremarkable. Her husband was 36 years old, and there was no family history of congenital malformations.

**Fig. 1:**
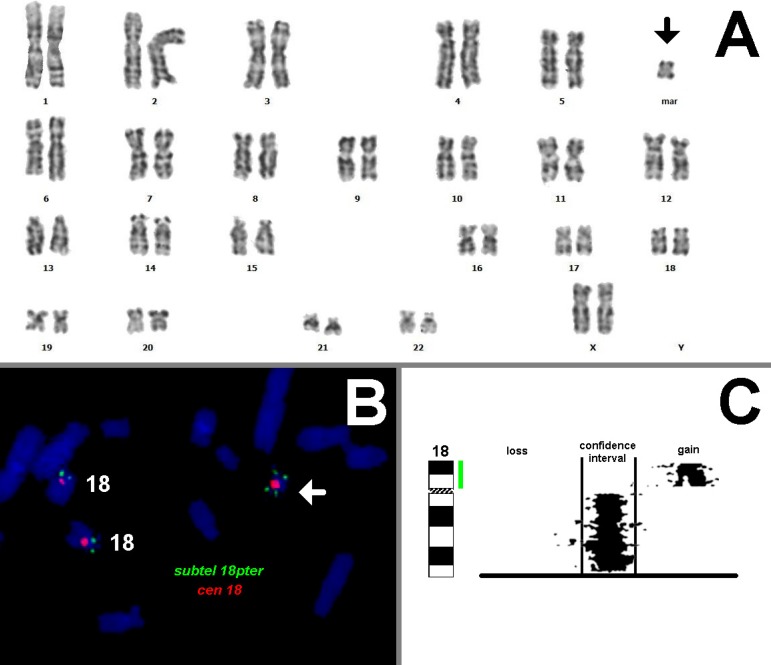
Cytogenetic analyzes. A: GTG-banding result after initial amniocentesis; the aberrant clone with the extra marker chromosome (arrow) is shown; B:FISH-result identifying the sSMC (arrow) as a i(18p); probes used were subtleomeric probe for 18pter (subtel 18pter) and centromirc probe for chromosome 18 (cen 18); C: Array-CGH-result showing the gain of copy numbers of the whole short arm of chromosome 18

Unexpectedly an abnormal karyotype of 47,XX,+mar dn[36]/46, XX[4] was found after GTG banding analyzes from two separate amniocyte cultures (Fig. 1A). C banding revealed a centeric marker and parental karyotypes were normal (results not shown). After genetic counseling, she underwent umblical cord blood sampling (cordocentesis) at 26 weeks of gestation and again an abnormal karyotype of 47,XX,+ mar dn[18]/46,XX[22] was found. Fluorescence in situ hybridization (FISH) using subtelomeric and centromeric probes (Abbott, USA) confirmed the karyotype results and identified the sSMC as an i(18)(pter- >q11.1: q11.1- >pter), in short I (18p) (Fig. 1B). Molecular karyotyping was performed using CYTOCHIP ISCA 8x60k whole genome oligo array version 2. An 18.4 Mb gain on chromosome 18p11.32q11.2 was confirmed, compatible with tetrasomy for the mentioned region (Fig. 1C).

**Fig. 2:**
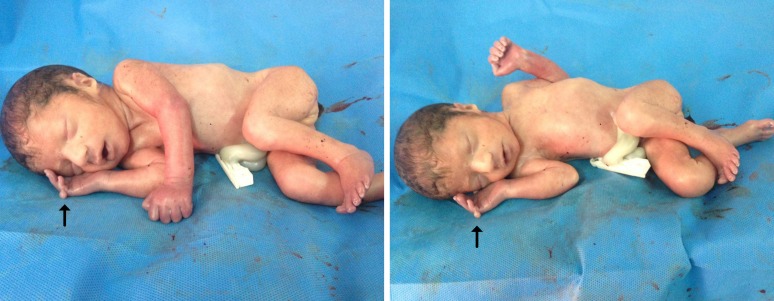
Photographs of the index patient after abortion. The extra finger on the right hand is highlighted by an arrow

The parents decided to terminate the pregnancy after genetic counseling and agreed in post mortem examination. Weight, length and head circumfe-rence were normal according to age of pregnancy and there was no indication for intrauterine growth retardation (IUGR). However, facial features included low anterior hairline, large philtrum, mild retrognathia and low- set posteriorly rotated malformed ears with prominent antihelix. In limb examination, joint contracture of the lower limbs and digital anomalies including long and narrow toes with clinodactyly of the 1^st^ and 5^th^ toes and postaxial polydactyly of one hand was seen (Fig. 2).

## Discussion

Among supernumerary isochromosomes, those derived from the short arm of chromosome 18 are the ones most commonly observed (2, 3). Tetrasomy 18p-syndrome due to sSMC-formation is *de novo* in the overwhelming majority of the reported patients, like in the here presented one. However, exceptionally there are also familial cases (3, 10). The latter supports again what was mentioned before, i.e. i(18p)-cases may have a high clinical variability (3, 5, 6). This makes the *de novo* occurrence of an i(18p), especially when being present as a mosaic state, a considerable challenge in prenatal settings. In the case of i(18p) this could be even more substantial from this point of view that as we saw, sonographic signs and expected phenotypes can be so mild that they are overlooked. In addition, the fact that the present case had no remarkable echocardiogram made the problem worse, although this fits well to the fact that cardiac anomalies are present in only 12.3% of such patients (5, 6).

For comparable cases with normal sonography a cordocentesis has been regarded as a useful step to get further insights into the fetal situation. Chen et al. reported in a prenatally detected mosaic tetrasomy 18p, a cytogenetic discrepancy between amniocytes (mosaic) and cord blood lymphocytes (no i(18p) detectable) (11). After genetic counseling the pregnancy was carried to the term and a normal male baby was delivered. In another case Jung et al. did cordocentesis at 32 weeks of gestation because of congenital heart disease, IUGR, cardiomegaly, and imperforate anus observed in prenatal ultrasound, with tetrasomy 18p detected in karyotyping and FISH analysis (12).

What can be implied from our case and the other cases is that, *de novo* i(18p) can be considered as a special case in the sense that the major relevant phenotypes mentioned for it, i.e. feeding difficulties, abnormalities in muscle tone and developmental/ mental retardation, cognitive and behavioral characteristics, recurrent otitis media and seizures, are mostly postnatal (5, 6). This emphasizes the necessity to determine the nature of a *de novo* euchromatic marker chromosome, especially in cases with normal ultrasound result, and highlights that studying a second fetal tissue (e.g. cordocentesis) can help the parents to better understand the situation they are in.

## Conflict of interest

The authors declared no conflict of interest.
